# Opposing Association of Situational and Chronic Loneliness with Interpersonal Distance

**DOI:** 10.3390/brainsci11091135

**Published:** 2021-08-27

**Authors:** Nira Saporta, Dirk Scheele, Jana Lieberz, Fine Stuhr-Wulff, René Hurlemann, Simone G. Shamay-Tsoory

**Affiliations:** 1 School of Psychological Science, University of Haifa, Haifa 3498838, Israel; fstuhrwu@campus.haifa.ac.il (F.S.-W.); sshamay@psy.haifa.ac.il (S.G.S.-T.); 2 Division of Medical Psychology, Department of Psychiatry and Psychotherapy, University Hospital Bonn, 53105 Bonn, Germany; dirk-scheele@gmx.de (D.S.); Jana.Lieberz@ukbonn.de (J.L.); 3 Department of Psychiatry, School of Medicine & Health Sciences, University of Oldenburg, 26129 Oldenburg, Germany; rene.hurlemann@uol.de; 4 Department of Education and Psychology, Freie Universität Berlin, 14195 Berlin, Germany; 5 Research Center Neurosensory Science, University of Oldenburg, 26129 Oldenburg, Germany

**Keywords:** loneliness, chronic loneliness, interpersonal distance, COVID-19, social interaction, situational loneliness

## Abstract

Loneliness is a prevalent condition with adverse effects on physical and mental health. Evolutionary theories suggest it evolved to drive people to reconnect. However, chronic loneliness may result in a negative social bias and self-preservation behaviors, paradoxically driving individuals away from social interactions. Lonely people often feel they are not close to anyone; however, little is known about their interpersonal distance preferences. During COVID-19, many experienced situational loneliness related to actual social isolation. Therefore, there was a unique opportunity to examine both chronic and situational (COVID-19-related) loneliness. In the present study, 479 participants completed an online task that experimentally assessed interpersonal distance preferences in four conditions—passively being approached by a friend or a stranger, and actively approaching a friend or a stranger. Results show that high chronic loneliness was related to a greater preferred distance across conditions. Intriguingly, by contrast, high COVID-19-related loneliness was related to a smaller preferred distance across conditions. These findings provide further support for the evolutionary theory of loneliness: situational loneliness indeed seems to drive people towards reconnection, while chronic loneliness seems to drive people away from it. Implications for the amelioration of chronic loneliness are discussed based on these findings.

## 1. Introduction

Loneliness is a painful experience, representing a subjective evaluation of one’s social relations as either quantitatively or qualitatively lacking [1], or a chronic perception of social isolation [2]. While highly correlated with other concepts, such as depression and anxiety, loneliness was established as a separate construct [3,4]. The prevalence of loneliness is considerable (e.g., [5,6,7,8]) and it has been shown to have harmful effects on physical and mental health [9], including an increase in morbidity and mortality [10], increased risk for coronary heart disease and stroke [11], depression [12], and dementia [13]. The importance of tracking and acting against loneliness has gained public recognition, as reflected, for example, by the inclusion of loneliness measurements in the U.S. Health and Retirement Study (HRS) and the appointments of loneliness ministers in the UK and Japan.

From an evolutionary perspective, loneliness may have evolved as an aversive signal, which drives people to reconnect with others, in the same way that hunger drives people to search for food [14,15]. However, evolutionary theory also contends that chronic loneliness may result in a perception of the social environment as one that will not provide protection and help, which then activates neural, neuroendocrine, and behavioral responses geared towards self-preservation and survival [16]. Thus, paradoxically, loneliness may trap individuals in an impossible situation, in which they crave connectedness but at the same time turn away from it.

Indeed, in a recent study, lonely participants reported less craving and showed a reduced midbrain response for social contact in response to positive social cues after isolation [15]. Loneliness was associated with further functional differences, as lonely people showed greater activity in brain areas related to visual attention in response to negative social stimuli [17] and reduced reward-related activity in response to pleasant social pictures [18]. Blunted reactivity of the right insular cortex to emotional faces was found to mediate subjective isolation stress [19]. Loneliness was also linked to structural changes in brain areas related to social cognition, empathy, theory of mind, and social alignment, which suggests potentially impaired connectivity in broad neural circuits [20,21,22]. The right prefrontal cortex and the right insular cortex respond to social exclusion and lesions to these regions were significantly associated with decreased loneliness scores, suggesting that these regions need to be intact to perceive loneliness [23]. See Reference [24] for a recent review.

Lonely people often complain about not having anyone “close to them”. They rate their feelings of connection to close others lower than non-lonely people [25] and also report less intimacy, comfort, and understanding, as well as more caution and distrust in their relationships [26]. When reflecting on the self or close others, lonely individuals show a reduced representational similarity between the self and other on a neural level [27] and they demonstrate a greater cognitive distance between the self and a friend [28]. However, does this mean they have different preferences regarding interpersonal space and distance? Interpersonal space is defined as “the area individual humans actively maintain around themselves into which others cannot intrude without arousing discomfort” [29]. When the expected interpersonal distance is intruded upon, people may feel threatened or anxious [30,31]. However, a closer interpersonal space preference is related to feeling more empathetic [32] and more comfortable with interpersonal emotional closeness [33]. There is evidence for individual trait-related preferences for interpersonal distance. For example, individuals with high social anxiety prefer greater distances [31], as do individuals with post-traumatic stress disorder (PTSD) [34] and high sensory sensitivity [35]. Individuals with autism spectrum disorder (ASD) demonstrate a greater variance in interpersonal distance preferences [36,37].

In general, the preferred distance from a stranger is greater than the preferred distance from a friend [38], and it seems that different neural circuits are involved in modulating distance from friends and strangers, with areas that are relevant to social interest and affiliation being activated when a friend is approaching as opposed to a stranger [35,39]. The difference between strangers and friends is particularly relevant to the discussion of loneliness. Lonely people may show a negative cognitive bias such that they expect social negative evaluation from their interactions [40], and therefore enter social interactions with strangers with motivations that are primarily focused on avoidance and security. Furthermore, higher levels of loneliness predicted greater reward-related brain activity in response to seeing close others compared to strangers, while at lower levels of loneliness there was no such difference [25]. The proposed interpretation of this finding was that lonely people do not view strangers as possible targets of social connection, and therefore they focus their ‘social craving’ on people they already feel close to.

If lonely people indeed crave more connection with their friends, it can be expected that they would prefer to be even closer to friends when compared to non-lonely individuals. Similarly, if lonely people indeed tend to avoid strangers, it can be expected that they would prefer a greater interpersonal distance from strangers when compared to non-lonely individuals. Indeed, a preference for a greater interpersonal distance from an unfamiliar person was found in highly lonely individuals [41]. In a questionnaire study, loneliness predicted preferences for greater interpersonal distance only within intimate space, but no differences were found for distance preferences from strangers and from friends [42].

Interpersonal distance also depends on whether one is approaching someone or is being approached. For instance, the hypothalamic peptide oxytocin (OT) significantly increased the preferred distance from a stranger but not from a friend who was approaching the participant [43,44]. Interestingly, the reversed pattern was found when the participant was the one approaching [45]. These findings suggest that one variation (passively being approached) activates the avoidance/threat system more, and the other variation (actively approaching) activates the approach/reward system more. Indeed, it was shown that being approached results in a greater distance than actively approaching [46,47]. However, no study to date has examined loneliness in this context. Therefore, the first aim of the current study was to characterize the preferred distance from friends vs. strangers in different approach conditions in chronic loneliness.

Since the onset of the COVID-19 pandemic, many reports concerning increased loneliness and its impact have emerged [48,49,50,51]. Unlike chronic loneliness discussed above, this loneliness is more situational in nature and was also accompanied by social distancing and, at times, complete isolation. As mentioned above, evolutionary models propose that transient loneliness is adaptive to the extent that it motivates social reconnection [4,52]. Indeed, it was shown that social exclusion motivates reconnection and predicts positive evaluation of future interaction partners [53], as well as increases attention to smiling faces [54]. In line with this is the finding that acute isolation led to the craving of social connection [15]. However, other studies show contrasting results. For instance, the experimental induction of state loneliness using hypnosis resulted in increased fear of negative evaluation, lowered self-esteem, and increased shyness, which would be expected to reduce the chance of a positive social interaction [4]. In a more recent study, state loneliness had complex effects, leading to both increases and decreases in subsequent social interaction [55]. Therefore, the second aim of the current study was to explore whether perceived situational loneliness that is related to COVID-19 impacts interpersonal distance preferences differently than chronic loneliness.

In the present study, we used a computerized interpersonal distance task, comparing responses to strangers and friends in both approach types (being approached and approaching). According to the view of chronic loneliness as inducing a self-preservation state, we hypothesized that lonelier individuals would have a general preference for greater interpersonal distances from strangers [41]. When comparing their reactions to friends, there were two competing hypotheses. If lonelier people crave more connection with close others, it can be expected that they would prefer to be even closer to friends. However, if lonelier people do not feel as close to their friends, it may be reflected in a preference for a greater distance from them.

In addition, as being approached seems to activate the threat system and approaching others seems to activate the reward system, it was hypothesized that distances would be greater in the passively being approached condition, and even more so for the lonely individuals.

Finally, we examined situational COVID-19 loneliness. As conflicting results exist about the relation between situational loneliness and social interaction, there were two possible hypotheses. Building on studies showing that situational loneliness, as induced by the COVID-19 pandemic, results in heightened needs for connection, preferred distances are expected to be smaller. If, however, it results in negative perceptions relevant to social interaction, preferred distances will be greater. Potential interactions between chronic and situational loneliness were also explored.

## 2. Materials and Methods

### 2.1. Participants

Participants were recruited on Amazon MTurk. MTurk had been previously validated for use in research [56]. All participants had a “master” status, a qualification that signifies an ability to consistently submit high-quality results, as indicated by approval rates and other related factors. Out of a total of 564 participants, 30 participants encountered technical issues accessing the interpersonal distance task (mostly due to use of old operating systems). Additionally, 31 participants were excluded from analysis due to partial data submission and 24 participants were excluded from the analysis due to an extremely fast response time in the interpersonal distance task, which signifies inattention to the task itself (mean response time across conditions in the task = 2.68 s, SD = 0.70 s, participants were excluded if mean response time across all conditions was more than two standard deviations lower than the mean). The final sample therefore included 479 participants (238 males, 241 females; age 24–78, mean age 43.13, SD = 10.87). Participants were recruited from all countries and the final sample included participants from the U.S. (370 participants), India (93 participants), the UK (2 participants), and 1 participant from each of the following countries: Afghanistan, Brazil, Canada, Colombia, Hong Kong, Italy, Kenya, Mexico, Singapore, Sri Lanka, Thailand, Republic of North Macedonia, United Republic of Tanzania, and Venezuela.

All participants passed basic attention checks (they were required to select a specific response to assure they were reading the questionnaire’s text). Participants who completed the study, or participants that could not complete the study due to technical issues, received USD 3.

All participants gave informed consent prior to participating in the study. The study was approved by the University of Haifa Ethics Committee.

### 2.2. Procedure

The study was conducted online via the Qualtrics survey platform (https://www.qualtrics.com/, (accessed date: 17 June 2021)) and Pavlovia (https://pavlovia.org/, (accessed date: 17 June 2021)).

After recruitment via Mturk, participants received the Qualtrics questionnaire URL. There, they provided informed consent, responded to demographic questions (sex, age, country), the UCLA loneliness scale [57], and a question regarding COVID-19 loneliness. They were then provided with a completion code for the first part of the study and the URL to the second part of the study, hosted in Pavlovia. In Pavlovia, participants entered the first part completion code and then performed the interpersonal distance task (see the Measures Section below). Upon completion of the task, they received a second code, which they had to enter back in Mturk to receive the payment. Codes were cross-checked and participants were paid only if they completed both parts.

### 2.3. Measures

#### 2.3.1. Interpersonal Distance Task

**Figure 1:**
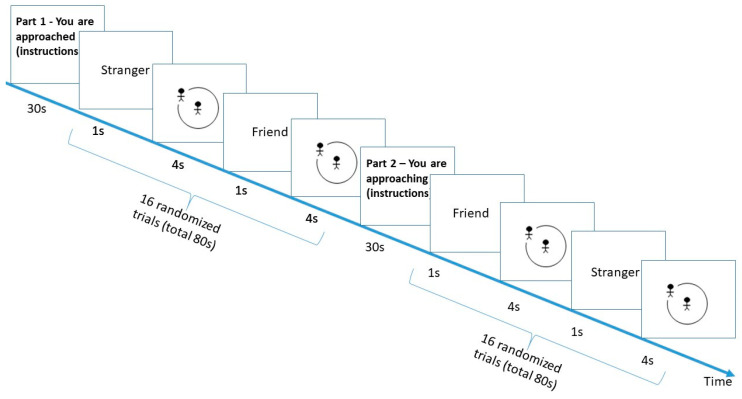
Interpersonal distance task.

To measure interpersonal distance preferences in response to virtual persons, a pen and pencil paradigm called the Comfortable Interpersonal Distance (CID) task was developed [58]. More recently, a computerized version of this task was developed in our lab [31,37]. The current computerized animated version was built using PsychoPy 3.0 [59] and was hosted online on Pavlovia.org. The participants were instructed to imagine themselves at the center of a room represented on a computer screen as a circle. They were then requested to respond to a line-figure protagonist animation (either a stranger or a close friend) approaching the center of the room. The animation stopped either at the end of the trial (when reaching the center), or when the participant pressed the space key, which stopped the figure’s approach at the preferred distance (see Figure 1). This computerized task was previously validated in our lab as reflecting differences in the preferred interpersonal distance in real life [31]. In a variation of this task, the participants are instructed to imagine themselves as the virtual person approaching another person (friends/stranger) at the center of the virtual room.

Each trial consisted of a slide that was presented for 1 s stating “Friend” or “Stranger”, followed by a presentation of the room and the approaching protagonist. The task was designed to assure maximal attention and participation. First, audio instructions were included in addition to the displayed text. Second, the maximum time of protagonist movement was 4 s. If the participant did not press the space key at all during the trial, a slide was presented verifying that the participant had indeed intended to select the minimal distance. This was done to avoid a situation where the participant would leave the computer and let the experiment run on its own. In addition, even if the participant chose to stop the protagonist quickly, the trial would still last the full 4 s. This was designed to prevent participants from pressing the space bar quickly regardless of the task in order to rush the experiment.

The participants were first presented with three training trials. They were then presented with a block of 16 trials, 8 for the friend and 8 for the stranger, and each protagonist appeared once in one of the following radii—0°, 45°, 90°, 135°, 180°, 225°, 270°, and 315°. The trials within the block were randomized.

The participants were then instructed to imagine themselves as the approaching figure, approaching either a stranger or a friend at the center of the virtual room. They were presented again with a randomized block of 16 trials, 8 for the friend and 8 for the stranger; each protagonist appeared once in one of the following radii—0°, 45°, 90°, 135°, 180°, 225°, 270°, and 315°.

Response times were recorded. A faster response time equals a greater preferred distance.

#### 2.3.2. UCLA Loneliness Scale

Participants completed the UCLA scale questionnaire version 3 [57]. The UCLA scale was initially developed in 1978 [60] and has since been revised twice to improve its validity and reliability. In the current version, the respondent is asked to rate the frequency of loneliness-related experiences. Some items refer to negative experiences, for example “How often do you feel left out?” and some items refer to positive experiences, for example “How often do you feel part of a group of friends?”. Each item is rated on a scale of 1 (never) to 4 (often), and after reversing the questions that relate to positive experiences, a total loneliness score (20–80) is calculated. The UCLA scale has high test-retest reliability [57,61] and it is often used to measure chronic, trait-like loneliness [27,62]. The mean score in the UCLA scale in the study was 42.84 (SD = 13.23) and the median score was 41.

#### 2.3.3. COVID-19 Loneliness

Participants were asked to indicate if there was a change in how lonely they have felt during the COVID-19 pandemic (1—a lot less, 2—a bit less, 3—no change, 4—a bit more, 5—a lot more). The mean score was 3.38 (SD = 0.94) and the median score was 4.

### 2.4. Experimental Design

A within-subject design was employed with the protagonist (stranger/friend) and the approach type (passive approach, i.e., participant being approached vs. active approach, i.e., participant approaching) serving as within-subject independent variables, and the level of loneliness and COVID-19 loneliness as between-subject independent variables. The dependent variables were the response times in each of the four conditions.

We employed a linear mixed effects (LME) analysis using R language [63], that allows for testing for interaction effects between two or more fixed factors and the slopes of continuous covariates in mixed designs, while accounting for the dependency between observations measured in the same participants. We employed an analysis to examine the effects of approach type and protagonist as within-subject fixed factors and the slopes of loneliness and COVID-19 loneliness as between-subject interaction terms on response time. We then employed the same analysis with sex (male/female) or with country as an additional interaction term. As most participants came from two countries (the U.S. and India), we excluded 16 participants from other countries in the analysis with country as an additional between-subject independent variable. In all models, subject was used as a random factor in order to account for the dependency in performance between the task conditions.

Additional statistical tests included *t*-tests and bi-variate Pearson correlations. Effect sizes were estimated using Cohen’s d. Bonferroni corrections were used for multiple comparisons.

Cronbach’s Alpha was calculated on the UCLA loneliness scale as a measure of its reliability.

All statistical analyses were performed using SPSS 25.0 or R language version 1.4.1106, using the LME4 package [64].

## 3. Results

**Figure 2:**
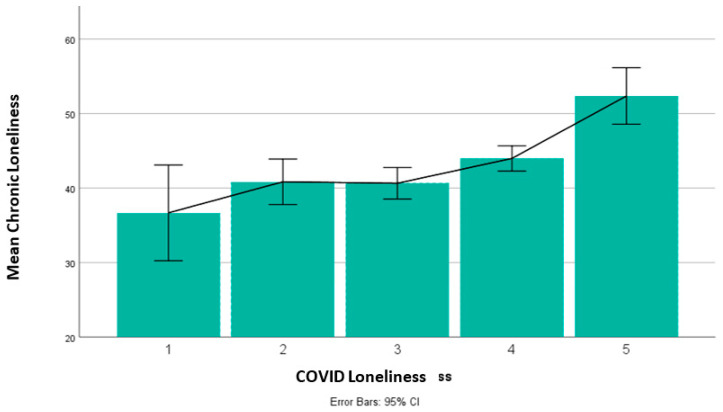
Chronic loneliness measured with the UCLA loneliness scale was higher in participants with higher COVID-19 loneliness.

The reliability of the UCLA Loneliness Scale was found to be excellent (Cronbach’s Alpha = 0.96). Chronic loneliness positively correlated with COVID-19 loneliness (r_(477)_ = 0.23, *p* < 0.001), see Figure 2. However, chronic and COVID-19 loneliness showed opposing associations with interpersonal distance.

In our first LME analysis, we examined the effects of approach type (participant being approached/participant approaching), protagonist (stranger/friend), chronic loneliness, and COVID-19 loneliness on the response time (with the subject as a random effect). Four models were constructed as follows: one examining main effects only, the second adding two-way interactions, the third also examining the three-way interactions, and the fourth examining all possible main effects and interactions. The predictive power of each model was compared to the preceding model using Type II Wald chi-square tests [63]. Results showed that the two-way model yielded a significantly stronger prediction power compared to the first model (χ^2^_(6)_ = 16.78, *p* < 0.05), whereas neither the three-way nor the four-way models were significantly better. Therefore, the second (two-way) model was used in further analyses.

**Figure 3:**
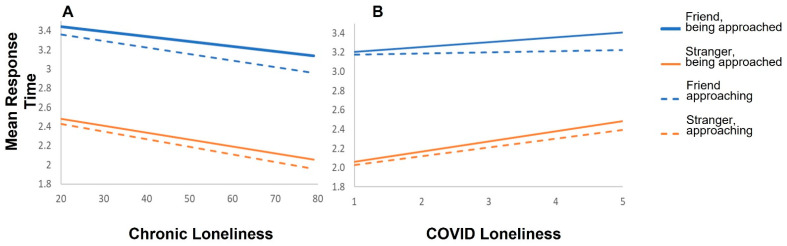
(**A**). Higher chronic loneliness is associated with a faster response time (preference for a greater distance) across all conditions. (**B**). The reverse pattern exists for COVID-19 loneliness. Higher COVID-19 loneliness is associated with a slower response time (preference for a smaller distance) in all conditions.

Analyzing the two-way model (Type II ANOVA) revealed significant main effects for the slope of loneliness (F_(1472.12)_ = 18.24, *p* < 0.001), the slope of COVID-19 loneliness (F_(1485.07)_ = 11.20, *p* < 0.001), approach type (F_(1,1434)_ = 21.33, *p* < 0.001), and protagonist (F_(1,1434)_ = 2319.85, *p* < 0.001). Across conditions, chronic loneliness as measured by the UCLA scale was negatively correlated with response times (r_(477)_ = −0.16, *p* < 0.001), translating to overall greater preferred distance for participants who had higher UCLA scores, while COVID-19 loneliness was positively correlated with response times (r_(477)_ = 0.11, *p* = 0.02), translating to a smaller preferred distance for participants with higher COVID-19 loneliness scores. These correlations were significantly different (z = 6.47, *p* < 0.001). See Figure 3. 

Being approached (across both protagonist conditions) resulted in a slower response (i.e., smaller distance) (M = 2.82 s, SD = 0.56 s) compared to actively approaching (M = 2.72 s, SD = 0.60 s), and response to friends was slower (i.e., smaller distance) across both approach types (M = 3.26 s, SD = 0.45 s) compared to strangers (M = 2.28 s, SD = 0.81 s). This result also serves as a validation of the task, as it is expected that the preferred distance from a stranger will be greater than from a friend. In addition, a significant interaction between protagonist and COVID-19 loneliness was found (F_(1,1434)_ = 11.83, *p* < 0.001). To further investigate the source of this interaction, we analyzed the effect of the protagonist while adjusting for the slope of COVID-19 loneliness with Bonferroni corrections in each condition separately. We found a significant effect only in the stranger condition (*t* = 4.37, *p* < 0.001, SD = 0.65) and not in the friend condition (*t* = 1.78, *p* > 0.05, SD = 0.65). No other main effects or interactions were significant.

Exploratory post hoc correlation analyses showed that chronic loneliness was negatively correlated with the distance from both the friend (r_(477)_ = −0.17, *p* < 0.001) and the stranger (r_(477)_ = −0.12, *p* = 0.007). The correlation was larger with regards to the distance from a friend; however, these two correlations were not significantly different (z = 1.16, *p* = 0.12).

Furthermore, we repeated the LME analysis, with country as an additional between-subject variable. Results showed that the two-way model yielded a significantly stronger prediction power (χ^2^_(10)_ = 28.19, *p* < 0.005). Similar main effects of chronic and situational loneliness, protagonist, and approach type were observed, as in the main analysis. In addition, a significant interaction between country and protagonist was found (F_(1,__1383)_ = 8.352, *p* < 0.005). In both countries, the difference between friend and stranger was significant, but the effect was stronger for participants from India (χ^2^_(1)_ = 560.68, *p* < 0.001, mean difference = 1.097) compared to participants from the U.S.: (χ^2^_(1)_ = 1686.79, *p* < 0.001, mean difference = 0.947). There were no other significant main effects or interactions with the country variable.

We then performed the LME analysis again, with sex as an additional between-factor variable. The results showed that the two-way model yielded a significantly stronger prediction power (χ^2^_(10)_ = 36.537, *p* < 0.001). Again, similar main effects of chronic and situational loneliness, protagonist, and approach type were observed as in the main analysis. In addition, we observed a significant main effect of sex (F_(1451.66)_ = 6.18, *p* < 0.05) and a significant interaction between sex and protagonist (F(_1,1383.97)_ = 16.94, *p* < 0.001). Men showed slower response times across all conditions (i.e., a smaller preferred distance) (M = 2.84 s, SD = 0.56) compared to women (M = 2.70 s, SD = 0.56). Follow-up *t*-tests showed that this sex difference was significant for strangers but not for friends. When being approached by strangers, females had a faster response time, i.e., a greater preferred distance (M = 2.21 s, SD = 0.84 s) compared to males (M = 2.42 s, SD = 0.81 s) (t_(1477)_ = 2.79, *p* = 0.005, Cohen’s d = 0.25, Bonferroni-correction for multiple comparisons). When approaching strangers, females also had a faster response time, i.e., a greater preferred distance (M = 2.13 s, SD = 0.85 s) compared to males (M = 2.37 s, SD = 0.82 s) (t_(1477)_ = 3.15, *p* = 0.002, Cohen’s d = 0.29, Bonferroni-correction for multiple comparisons).

## 4. Discussion

This study was designed to examine interpersonal distance preferences in individuals suffering from chronic or situational (specifically, COVID-19-related) loneliness. We used an online computerized task that experimentally measured interpersonal distance preferences in various conditions: (1) the participant is the one being passively approached or the one actively approaching, and (2) the protagonist approaching or being approached by the participant is a friend or a stranger.

Our initial hypothesis about chronic loneliness was confirmed, as loneliness was found to be related to a general preference for a greater interpersonal distance. This association was evident across all conditions and is consistent with previous findings based on stop-distance measurements in highly lonely individuals [41].

Even though situational, COVID-19-related loneliness was positively correlated with chronic loneliness, people who reported higher levels of COVID-19-related loneliness showed the reverse pattern: across conditions their preferred distance was shorter. This observation is in line with one of the competing hypotheses about situational loneliness, namely that it would result in increased social motivation for connection [15,52]. Likewise, elevated situational loneliness resulted in increases in prosocial behavior among older adults who were low in loneliness [65].

The study provided a unique opportunity to examine chronic and situational loneliness in the same population and support a model that differentiates their impact. The evolutionary theory of loneliness emphasizes the existence of conflicting motivations: on the one hand, lonely people are motivated to approach, in order to repair or replace social connections to assure self-preservation and, on the other hand, lonely people are motivated to be alert and avoid social threats, again in service of their self-preservation. Therefore, the evolutionary theory of loneliness (also referred to as ETL) predicts that loneliness will result in a conflict around approach and avoidance [16]. In this study, we were able to further deepen the understanding of this approach-avoidance conflict and differentiate conditions in which loneliness will ultimately result in more approach versus more avoidance behavior. Our findings support the notion that short-term, situation-related loneliness functions as a signal that promotes reconnection efforts. It appears that under these conditions, the approach motivation is stronger. However, when loneliness becomes chronic, and is not situation-specific, the avoidance motivation becomes more prominent and drives individuals away from social interaction.

The literature on approach and avoidance motivations demonstrates that chronic loneliness is associated with social avoidance motivations. Individuals with strong avoidance motivations and goals attempt to avoid negative social outcomes. However, despite their intentions, they actually experience increases in these negative outcomes, as avoidance motivation leads to attending more to negative stimuli [66]. It was suggested that social approach motivation is primarily linked to outcomes through exposure to positive social events, whereas avoidance social motivation is associated with reactivity to negative social events [67]. Studies found that lonely people show increased attention to negative social cues [68,69,70,71]. Lonely people also report more negative subjective evaluations of their relationships, as reflected by a lower level of satisfaction, as well as more reported conflicts and lower levels of closeness [72]. Such negative evaluations, according to this model of approach and avoidance, would strengthen the avoidance motivation further. Our findings support this model of approach-avoidance motivations, as it is quite likely that people who suffer from long-term chronic loneliness, with stronger avoidance motivations, have been exposed to less positive social events, and attend such positive social events less when compared to people who report loneliness due to a specific situation, such as COVID-19.

Our hypothesis with regards to the differences between being passively approached and actively approaching was not supported, as our findings point in the opposite direction. Being actively approached resulted in a smaller interpersonal distance preference when compared to actively approaching. One potential explanation is that COVID-19 specifically impacted active approach, as people were encouraged not to get too close to others in order to protect their own health as well as that of others. There is some preliminary evidence that the preferred interpersonal distance potentially increased during the COVID-19 pandemic [73,74]. Additional research is required to deepen the understanding of the dynamics related to interpersonal distance preferences following COVID-19.

Across all conditions, females preferred a greater distance, and this sex difference was more pronounced for strangers than friends. It should be noted that we did not specify whether the protagonist was male or female and the participants could choose to imagine either. Prior literature on interpersonal distance had identified sex differences and, in general, it was claimed that females maintain smaller interpersonal distance during interactions [75,76,77]. In many studies, no significant differences between males and females in distance preference were found [78,79]. However, and most relevant to the current study, it was shown that, while female friends stood closer together than male friends, female strangers maintained a greater distance compared to male strangers [80]. Therefore, it appears that the specific conditions (e.g., whether interacting with friends or strangers) are critical for gender/sex differences in interpersonal distance preferences.

One limitation of this study is that it was conducted online with a recruitment process that utilized Amazon Mturk. Mturk has been validated for use in academic research in the past [56], and numerous measures were taken in order to assure the validity and reliability of the data collected. These include requiring a “master” status from all participants, designing the questionnaires with attention checks, and designing the interpersonal distance task in a way that would prevent participants from letting it run in the background without attending to it, or from clicking too fast in order to advance it without attending to it. Participants who showed inattention were not included in the analysis and the UCLA questionnaire showed excellent internal consistency. That said, future research is required to also confirm the findings of this study in a face-to-face setting, potentially using additional tasks that measure interpersonal distance preferences.

Another limitation of this study was that the measurement of COVID-19 loneliness was based on a single question. While many studies that examine situational loneliness rely on a single question to assess it (e.g., [62,81]), it would be preferable to measure it using a more robust assessment tool in future studies. In addition, COVID-19-related loneliness is a specific type of situational loneliness. Additional research is required to confirm the findings of this study across different types of situations that induce situational loneliness.

## 5. Conclusions

In this study, chronic loneliness was associated with a preference for a greater interpersonal distance. Although situational, COVID-related, loneliness was correlated with chronic loneliness, it had the opposite association with interpersonal distance preferences. This study supports the evolutionary theory of loneliness, according to which transient and situational loneliness promotes social interaction, but chronic loneliness drives people suffering from it away from reconnection. 

The findings of this study have both theoretical and clinical implications. On the theoretical level, they accentuate the requirement to carefully differentiate chronic and situational loneliness in academic studies. Although correlated, they represent very different human experiences and can even result in contrasting effects. On the clinical level, the study demonstrates that people who suffer from chronic loneliness may fail in initially approaching others, even in everyday situations, and even with people they already feel close to. Clinical interventions designed to ameliorate chronic loneliness should consider that even simple, potentially unconscious social gestures, such as maintaining the proper interpersonal distance from one’s friends, may be impaired among lonely people and in general work towards reducing the negative biases they experience in social interactions.

## Data Availability

The data of this study is available in the following URL: https://github.com/SANSlabHaifa/interpersonal-distance-loneliness, (accessed date: 24 August 2021).
